# Suture Repositioning for Subluxation of a Toric-Type Single-Piece Multifocal Intraocular Lens

**DOI:** 10.7759/cureus.89566

**Published:** 2025-08-07

**Authors:** Tsuyoshi Mito, Haruka Tsuruoka, Sayaka Omura, Motoki Chinen, Hiroshi Sasaki

**Affiliations:** 1 Department of Ophthalmology, Kanazawa Medical University, Uchinada, JPN

**Keywords:** lens subluxation, multifocal intraocular lenses, post cataract surgery, suture fixation, toric intraocular lens

## Abstract

Intraocular repair for subluxation of a multifocal intraocular lens (MFIOL) can be challenging, especially in toric models. Herein, we report a case of a 43-year-old man with a subluxated toric-type single-piece trifocal intraocular lens (IOL) with a C-loop that was spared using suture repositioning. Pars plana suturing was performed using the haptic externalization technique, and the tram-track suture technique was employed to flatten the tilted toric MFIOL. We also evaluated the postoperative toric axis and IOL position using anterior segment optical coherence tomography (AS-OCT) and a wavefront aberration analyzer. Postoperative AS-OCT revealed a 0.51 mm decentration and 8.7° tilt. The wavefront analysis revealed that the toric axis alignment was <5° from the target axis. The postoperative refractive error and residual astigmatism were minimal; the visual acuity at different distances was acceptable.

## Introduction

Intraocular lens (IOL) subluxation can occur after cataract surgery, with probabilities reported by Pueringer et al. [1] of 0.1% at 10 years and 0.7% at 20 years. If the subluxated IOL is a toric type of multifocal intraocular lens (MFIOL), it can present challenges for the surgeon. In general, even with monofocal IOLs, decentration and tilt can have an effect, such as an increase in higher-order aberrations [2,3]. However, the effects of decentration and tilt on MFIOLs may be more severe than those on monofocal IOLs because the complex structure of the optic, which provides excellent visual function but reduces contrast sensitivity [4,5]. Additionally, toric IOLs generally lose approximately 30% of their ability to correct astigmatism when 10° of axial misalignment occurs [6,7]. Therefore, it is challenging to reuse subluxated toric MFIOLs and refix them intraocularly. Herein, we report a case of subluxation of a single-piece toric MFIOL with C-loop treated with suture fixation. We also evaluated the postoperative toric axis and IOL position using anterior segment optical coherence tomography (AS-OCT) and a wavefront aberration analyzer.

## Case presentation

**Figure 1:**
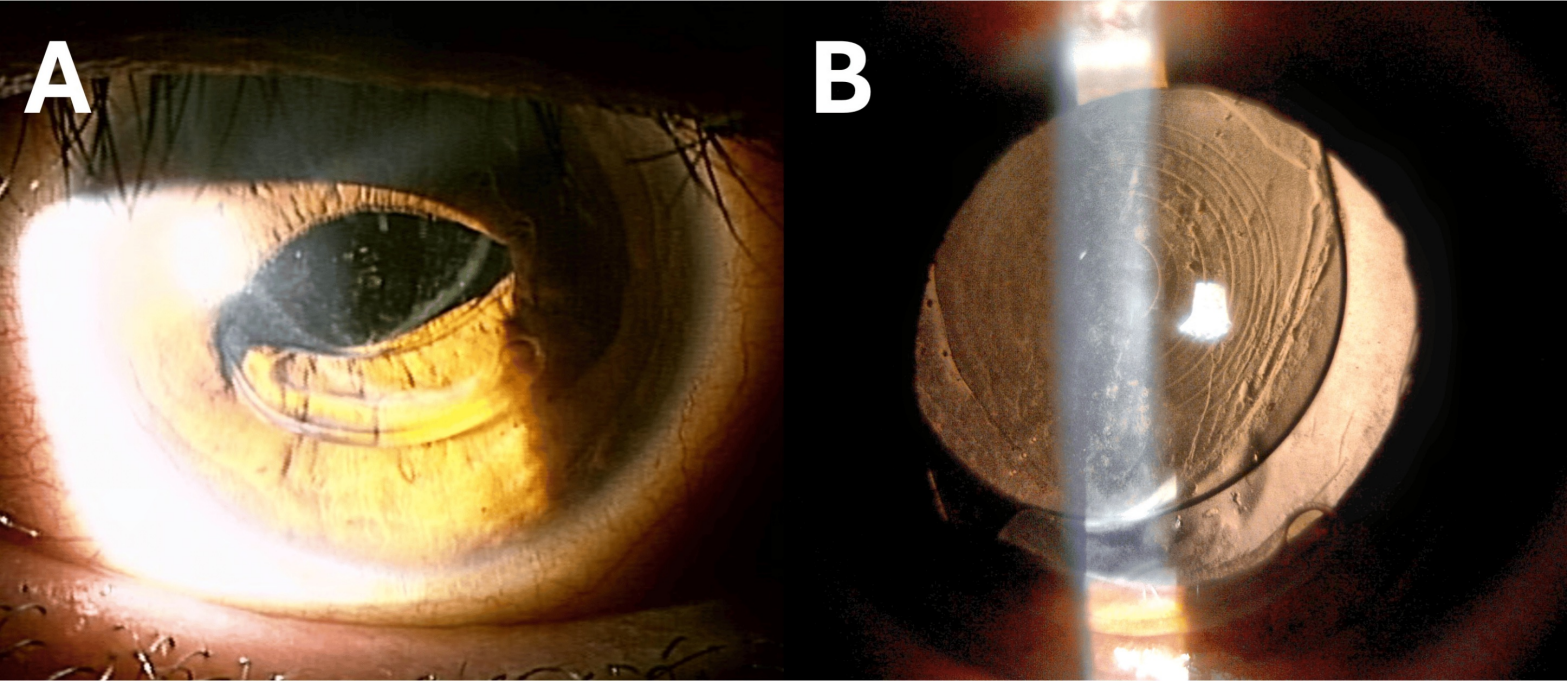
Preoperative photographs of the left eye anterior segment Before mydriasis, dislocation of the lower haptics into the anterior chamber was observed (A). Decentration of the multifocal intraocular lens (MFIOL) was observed under mydriasis, and toric axial marks are recognized at the one and seven o’clock positions (B)

The patient was a 43-year-old man. The referring physician informed us that he underwent cataract surgery in the left eye two years ago and had a toric type of trifocal IOL (AcrySof IQ PanOptix Toric Model TFNT30; Alcon Laboratories, Fort Worth, TX, USA), fixed intraocularly with a target axis of 55°. The patient did not have problems for a while after the surgery; then, suddenly, he complained of difficulty with vision, visited his primary doctor, and was diagnosed with IOL subluxation. At the time of referral to our department, examinations determined a corrected visual acuity of 10/20, intraocular pressure of 16 mmHg, and subluxation of the inferior haptics into the anterior chamber (Figure 1). 

**Figure 2:**
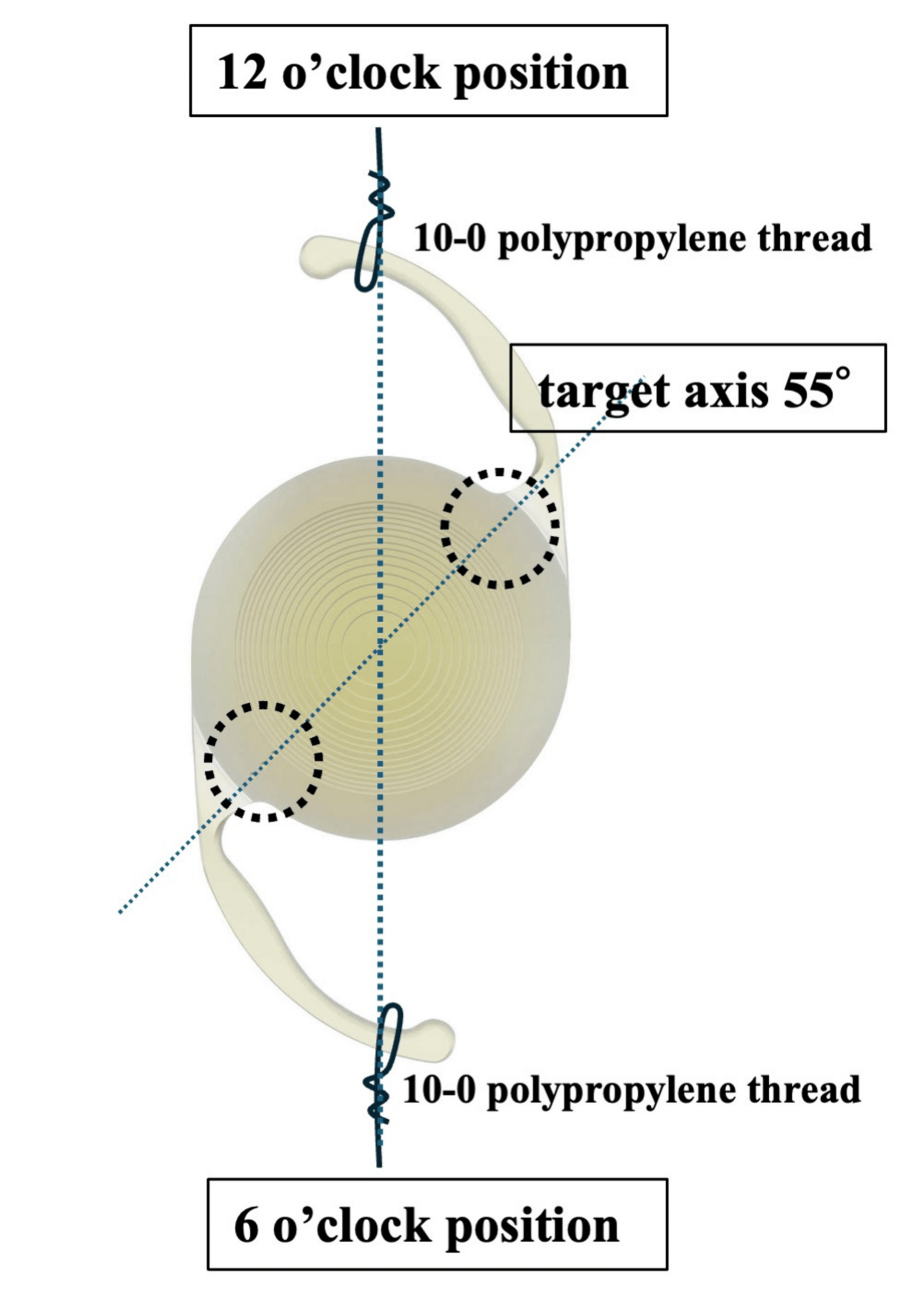
Image of toric axis alignment. Since the target axis of fixation for single-piece toric multifocal intraocular lens (MFIOL) was 55°, the 6 and 12 o’clock positions are designated as the haptics suture points.

**Figure 3:**
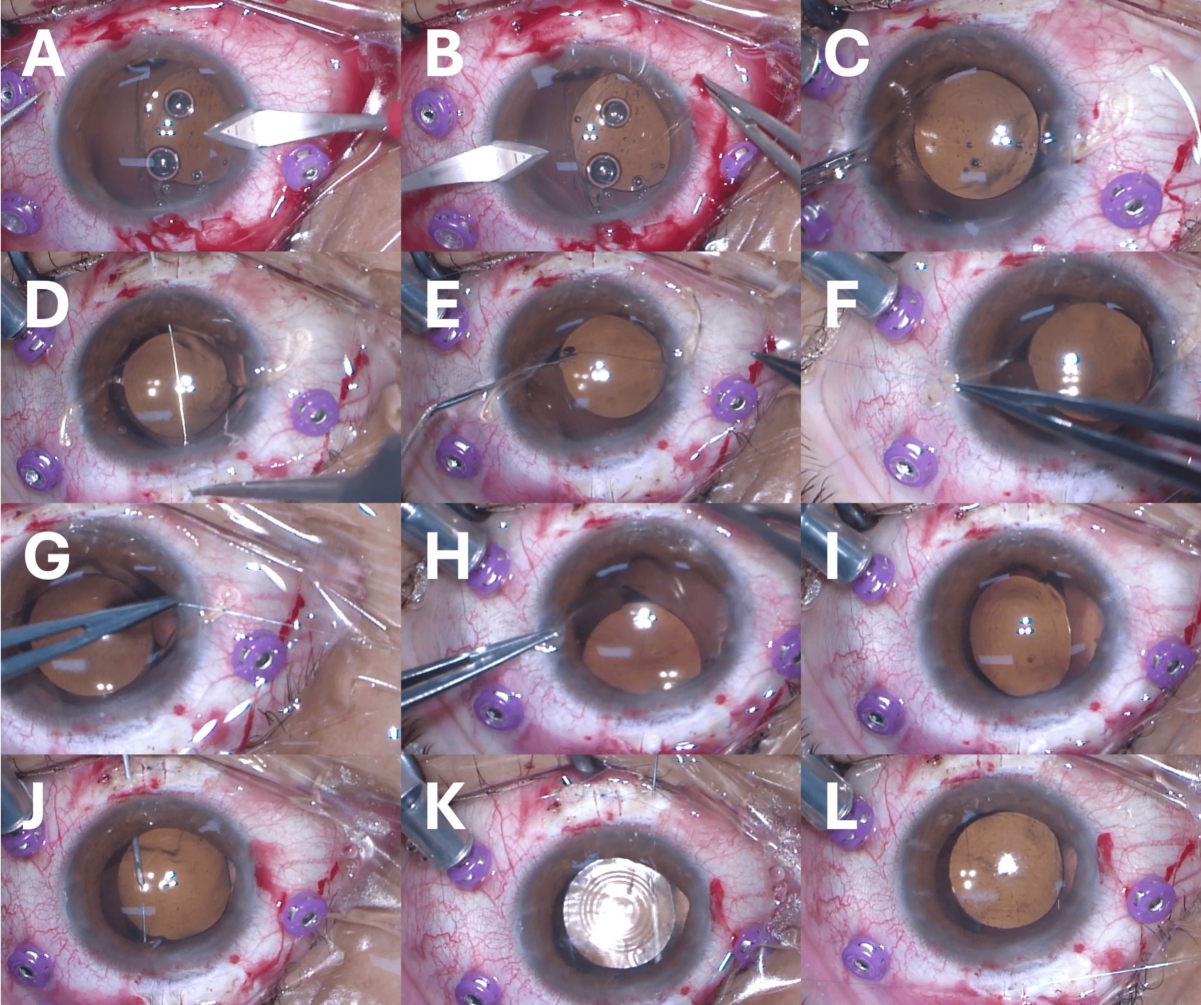
Intraoperative photographs Corneal limbal incisions are made at the three and nine o’clock positions (A, B), and the subluxated intraocular lens (IOL) is moved over the iris, and the haptics are extracted from the eye (C). A 10-0 polypropylene thread with a straight needle is threaded 3 mm posterior to the corneal limbus (D), the needle is pulled out of the eye, and the 10-0 polypropylene thread is cut and tied to the haptics (E-G). The haptics are replaced in the eye (H), and the IOL is moved to the posterior chamber and sutured to the pars plana, which causes IOL tilt (I). A 10-0 polypropylene thread is passed through the anterior (J) and posterior (K) surfaces of the IOL optics and tied to flatten the IOL (L)

Surgery was performed using the haptic externalization technique [8]. The suture location of the haptics to the sclera was planned at six and 12 o’clock positions, with the target fixation toric axis of the MFIOL at approximately 55° (Figure 2). The VERION image-guided system (Alcon Laboratories, Fort Worth, TX, USA), an intraoperative digital marker, was used for toric MFIOL axis alignment. First, anesthesia (subtenon 2% xylocaine) was administered. Second, a 27-gauge trocar was placed at the pars plana for subsequent vitrectomy. Incisions approximately 2 mm were made in the corneal limbus at the three and nine o’clock positions (Figures 3A, 3B). The subluxated toric MFIOL was moved over the iris, the lens capsule was separated from the IOL and removed, and both sides of the haptics were extracted from the eye through the corneal limbal incision (Figure 3C). After vitrectomy, a 10-0 polypropylene thread with a straight needle was inserted 3 mm posterior to the corneal limbus at the 12 o’clock position, threaded through the back of the IOL, and then the needle was removed from the eye at the six o’clock position (Figure 3D). The intraocular 10-0 polypropylene thread was extracted from the eye through the corneal limbal incision and cut; the 10-0 polypropylene thread was sutured to the haptics, and the haptics were replaced in the eye (Figures 3E-3H). Then, the IOL was moved to the posterior chamber, sutured, and fixed to the pars plana. At that time, the IOL tilted because of suture traction (Figure 3I). Therefore, the tram-track suture technique [9] was used to correct the IOL tilt. Initially, the 10-0 polypropylene thread with a straight needle was inserted into the eye through the superior sclera and threaded through the anterior surface of the three o’clock optic, which was tilted anteriorly, and the 10-0 polypropylene thread exited the eye through the inferior sclera (Figure 3J). Then, the 10-0 polypropylene thread was threaded back into the eye from the inferior sclera, and the needle was removed from the superior sclera, passing behind the optical area of the IOL at the nine o’clock position that was tilted backward (Figure 3K). Finally, the IOL tilt was flattened by ligating the 10-0 polypropylene thread (Figure 3L).

**Figure 4:**
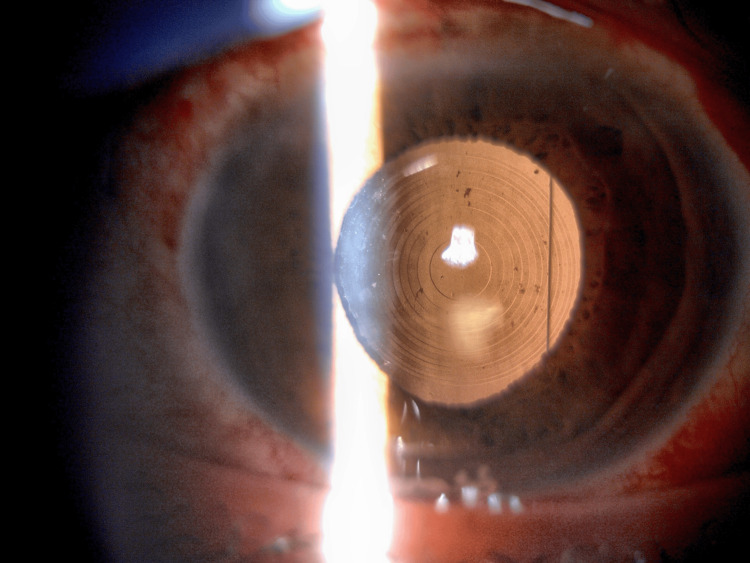
Postoperative anterior segment photograph within one week after surgery Minimal decentration of the multifocal intraocular lens (MFIOL) and 10-0 polypropylene thread with the tram-track suture technique is observed under mydriasis. The toric axial mark can be recognized at approximately 60°, close to the preoperative target

**Figure 5:**
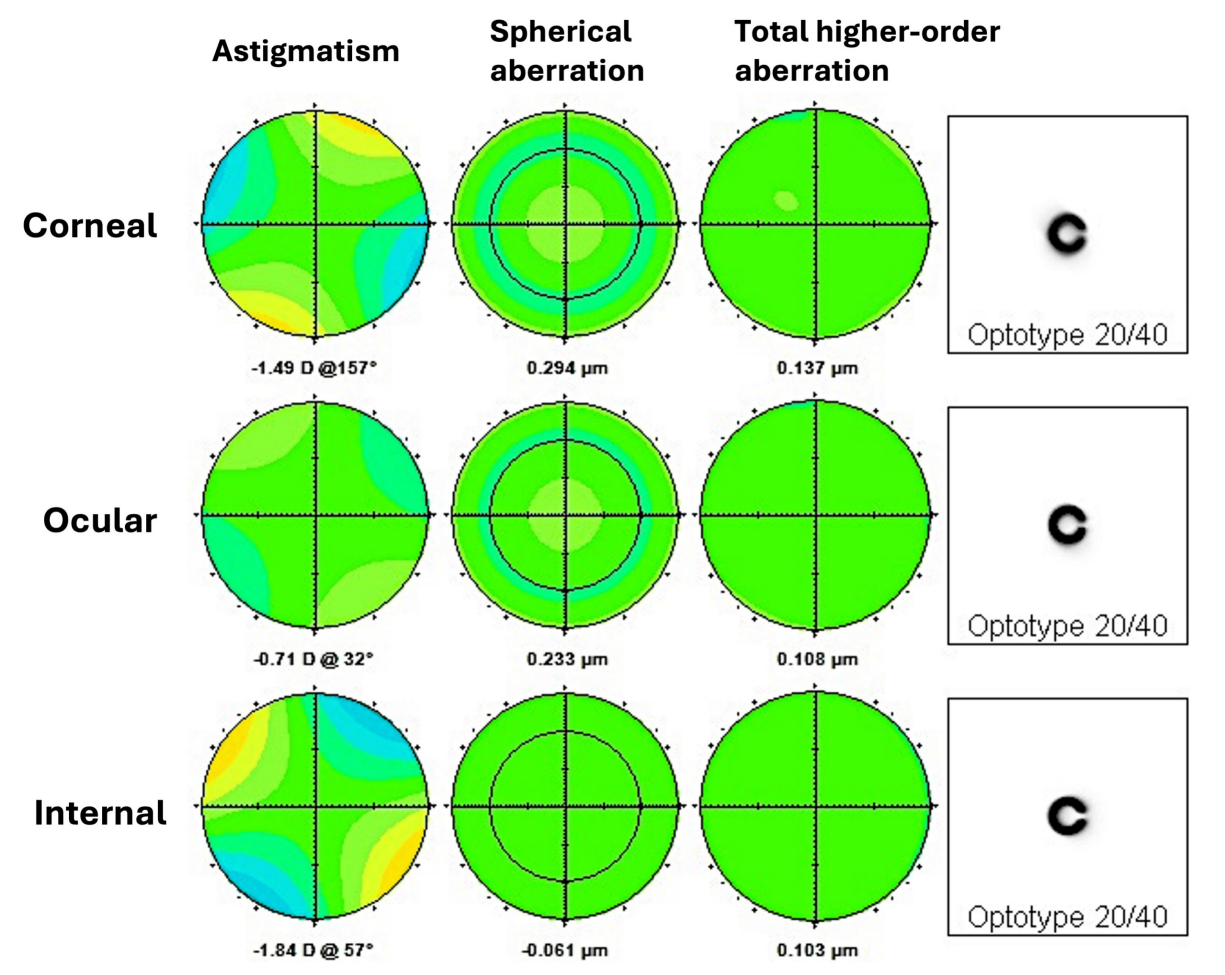
Postoperative wavefront aberration analysis at one month postoperatively The internal aberrations attributed to the toric multifocal intraocular lens (MFIOL) cancel out the corneal aberrations and reduce the ocular aberrations. Internal aberration is indicated by an astigmatic axis of 57°

**Figure 6:**
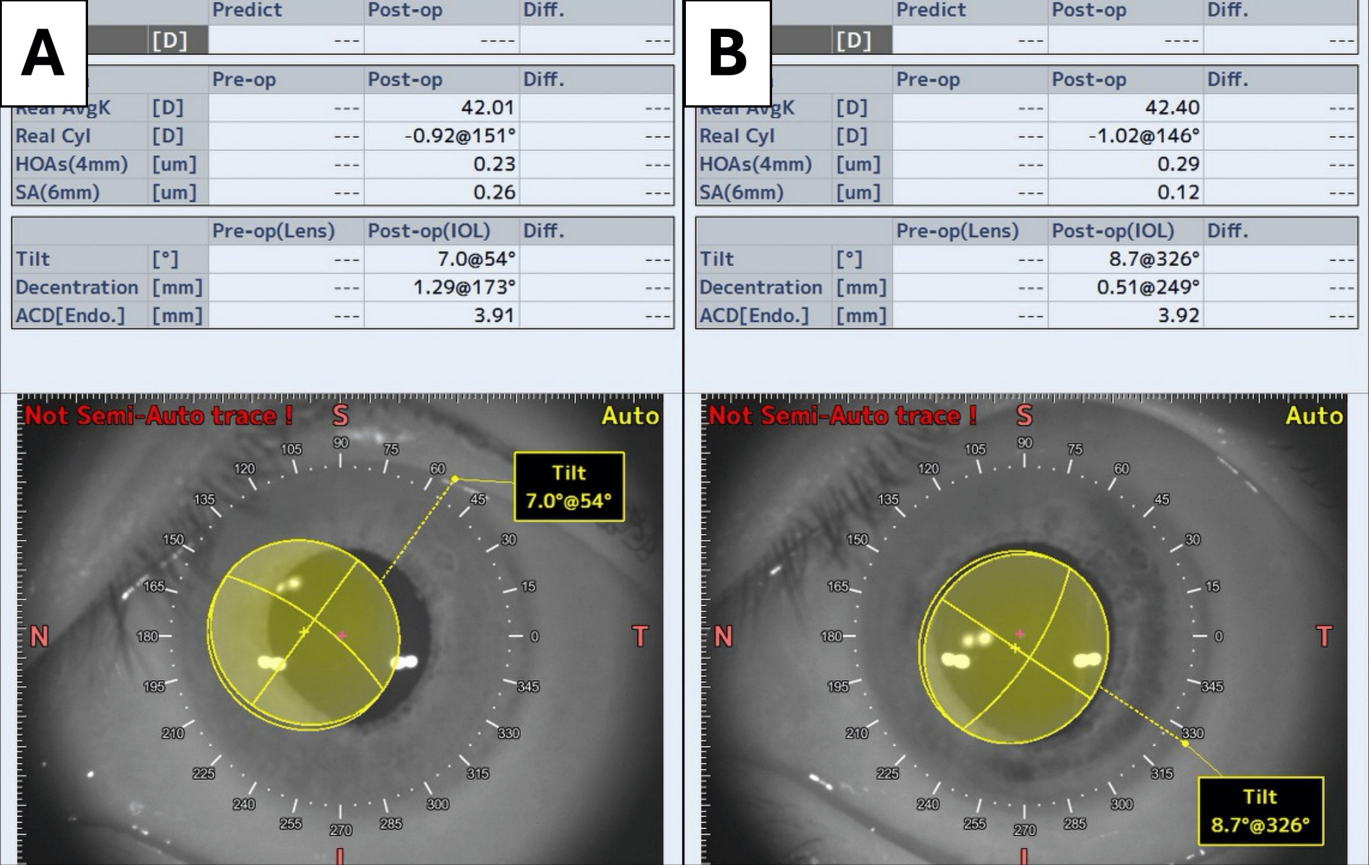
Anterior segment optical coherence tomography images at 1 month postoperatively Preoperatively, the multifocal intraocular lens (MFIOL) has a tilt of 7.0°, a decentration of 1.29 mm, and an IOL depth of 3.91 mm (A). Postoperatively, the tilt is 8.7°, the decentration is 0.51 mm, and the IOL depth is 3.92 mm (B)

**Figure 7:**
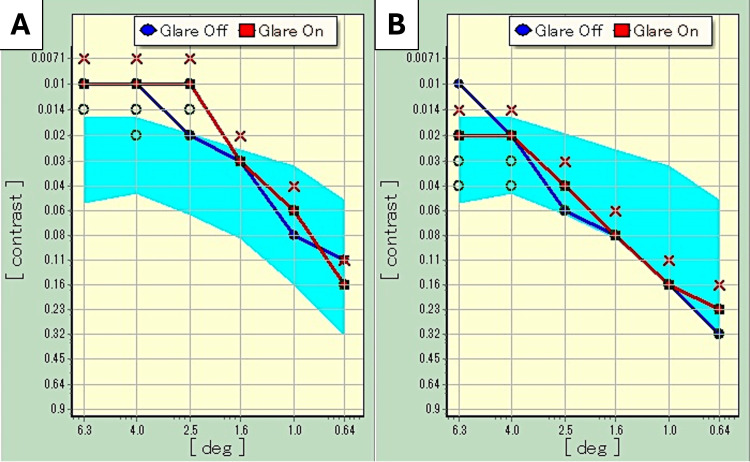
Contrast sensitivity function at one year postoperatively Results are within the normal range. Measured under photopic condition (A) and mesopic condition (B). Blue areas are the normal range for adults aged 40 to 60 years

The postoperative decentration of the IOL was small, and the toric axial mark could be recognized at approximately 60°, close to the target axis (Figure 4). The fixed axis of the toric MFIOL was estimated to be 57° based on the analysis of internal aberration using the wavefront aberration analyzer (KR-1W, TOPCON, Tokyo, Japan) (Figure 5). The AS-OCT (CASIA2, TOMEY, Nagoya, Japan) analysis showed that the preoperative IOL tilt, decentration, and depth were 7.0°, 1.29 mm, and 3.91 mm, respectively (Figure 6A), while the postoperative IOL tilt, decentration, and depth were 8.7°, 0.51 mm, and 3.92 mm, respectively (Figure 6B). Contrast sensitivity function measured using CGT-2000 (Takagi Seiko Co., Ltd., Tokyo, Japan) at one year postoperatively was within the normal range for age-matched controls (Figure 7). Postoperative uncorrected visual acuity was 20/25 or better at all tested distances from 30 cm to 5 m, with best distance acuity reaching 20/20. The corneal endothelial cell count was 3038 cells/mm^2^ preoperatively. Postoperatively, the number was 2863 cells/mm^2^, showing no significant decrease. Subjective severity of glare and halo was assessed. Glare and halo intensity is graded as none, mild, medium, or severe. The patient stated that the glare and halo were mild; he was aware of some photic phenomena, although they were not bothersome. A questionnaire was administered, which included a question about whether postoperative visual acuity was very good, good, normal, or poor for distance-, intermediate-, and near-vision, respectively; the patient indicated that distance-vision was very good, intermediate-vision was good, and near-vision was also good. At one year postoperatively, there were no complications and no significant changes in AS-OCT findings.

## Discussion

Most surgeons are concerned about the suturing fixation of a subluxated MFIOL, especially toric models. The reason for this is obvious: good postoperative visual acuity with toric MFIOLs requires less postoperative refractive error, residual astigmatism, decentration, and tilt. Reports on the intraocular suture fixation of subluxated MFIOLs are few, particularly with single-piece toric models [10-12]. Mahmood et al. [10] and Park et al. [11] reported suturing the FineVision toric IOL (PhysIOL, Liege, Belgium) with a technique utilizing the unique double C-loop haptics of that IOL. Eom et al. [12] sutured single-piece toric MFIOLs with the conventional C-loop using a special piercing method in which 6-0 polypropylene thread penetrated MFIOL optics. However, in those reports, there are no descriptions or evaluations of postoperative toric axis or IOL position. Therefore, it is unclear whether the toric MFIOL was fixed on the intended axis, and it is difficult to determine whether these techniques are appropriate as a method of reposition and fixation of subluxated single-piece toric MFIOLs. In this case, we used the haptic externalization technique [8] and the tram-track suture technique [9]; the postoperative AS-OCT and wavefront analysis showed that the toric IOL axis and position were favorable, suggesting that this technique is suitable as a method of repositioning a subluxated toric MFIOLs. 

In the present case, the subluxated MFIOL was a toric model; therefore, it had to be aligned with the target axis during suture fixation. We were able to achieve accurate astigmatism axis alignment because we performed sufficient preoperative simulations to determine the haptic suture location to the sclera. Specifically, since the angle between the tip of the lens haptics and the toric lens axis mark was determined to be approximately 40° to 50° in the image simulation, it was considered that the postoperative toric lens axis could be placed closer to the target of 55° if the toric MFIOL was sutured in the six and 12 o’clock directions. The use of the intraoperative digital marker VERION is considered one of the reasons for high toric axis alignment accuracy in this case. If an intraoperative digital marker is not available, the conventional method of marking corneal ring reference points in the horizontal and vertical axes with a pen or instrument with the patient in a sitting position is required before surgery. In such cases, intraoperative toric axis alignment accuracy is likely to be decreased.

IOL suturing techniques differ depending on the suture position, such as ciliary sulcus suturing [13] and pars plana suturing [14]. For ciliary sulcus suturing, the distance between the diagonals of the ciliary sulcus is approximately 11 mm, and the IOL haptics are fixed in the sulcus between the back of the iris and the anterior edge of the ciliary process, resulting in stable fixation with less decentration and tilt compared with pars plana suturing. However, the depth of the IOL fixed in the ciliary sulcus is 0.73 mm more anterior than that in the capsular bag fixation, which results in a refractive error of 0.5 diopters [15]. We considered that a myopic shift of 0.5 diopters with an MFIOL would have a significant influence on postoperative visual acuity. Therefore, in this case, we performed pars plana suturing, which is closer to the IOL depth in normal capsular bag fixation. By contrast, for pars plana suturing, the distance between the diagonals of the pars plana is approximately 13 mm. Soft acrylic single-piece IOLs are not sufficiently long, and the tension of the 10-0 polypropylene thread ligated to the haptics can cause IOL torsion, leading to decentration and tilt. In the present case, when the single-piece MFIOL was sutured to the pars plana, severe tilt due to IOL torsion occurred. Therefore, the tram-track suture technique [9] was added, and the IOL postoperative tilt was reduced to <10°. MFIOLs and toric IOLs tend to cause an increase in higher-order aberrations and a decrease in contrast sensitivity when a tilt of 5° or more occurs [16]. The postoperative tilt in the present case was 8.7°, which may have a significant impact on postoperative visual function. However, the contrast sensitivity was within normal range, and an increase in higher-order aberrations was not observed. No postoperative complications, such as intraocular inflammation or increased intraocular pressure, occurred because there was no contact between the IOL and the iris [17,18]. Careful follow-up is necessary because uveitis-glaucoma-hyphema syndrome [19,20] may develop in the future due to contact between the 10-0 polypropylene thread and the iris undersurface.

Certain important points should be considered for avoiding decentration during the suturing of a single-piece IOL with a C-loop. First, the location of the 10-0 polypropylene suture to the sclera must be exactly 180° to the contralateral side of the sclera. Second, the 10-0 polypropylene thread tied to the haptics on both sides of the IOL must be equally distant from the tip. Third, the haptics knot should be placed on the outer edge of the haptics. Fourth, if IOL decentration remains noticeable, it is necessary to check for residual vitreous or lens capsule around the IOL. Finally, these delicate techniques for tying 10-0 polypropylene thread to haptics cannot be performed within the anterior chamber or vitreous cavity; they are possible with the haptic externalization technique because the haptics are placed outside the eye.

## Conclusions

Subluxations associated with toric-type single-piece MFIOLs with a C-loop are expected to increase in the future. The findings of this case suggest that combining conventional haptic externalization and tram-track suture techniques may be effective for repositioning subluxated toric MFIOLs. Further studies involving more patients are needed to confirm the consistency and long-term outcomes of this technique.
